# A clinical study of thread carpal tunnel release with a newly developed thread: A retrospective pilot study

**DOI:** 10.1371/journal.pone.0276630

**Published:** 2022-10-21

**Authors:** Jisoo Park, In Jong Kim, Hae-yeon Park, Dong jin Heo, Jae Min Kim

**Affiliations:** 1 Department of Rehabilitation Medicine, Incheon St. Mary`s Hospital, College of Medicine, The Catholic University of Korea, Seoul, Republic of Korea; 2 Department of Rehabilitation Medicine, Howareyou Rehabilitation Clinic, Seoul, Republic of Korea; 3 Department of Rehabilitation Medicine, Seoul St. Mary’s Hospital, College of Medicine, The Catholic University of Korea, Seoul, Republic of Korea; Aminu Kano Teaching Hospital, NIGERIA

## Abstract

**Introduction:**

Previous studies have shown that, thread carpal tunnel release (TCTR), an ultrasound-guided transverse carpal ligament (TCL) transection procedure through needle and thread, to be a safe and effective technique for carpal tunnel release, compared to an open and endoscopic technique. We developed a newly improved thread (Smartwire-01, 0.27mm in diameter, Korea). This pilot study was performed to propose the effectiveness of TCTR with Smartwire-01 compared to the commercial thread in clinical settings.

**Methods:**

A total of 22 TCTR procedures have been performed on 19 patients by one physiatrist during a 42-month period. The diagnosis of carpal tunnel syndrome was based on standard clinical criteria including electromyography (EMG). Patients were divided into two groups, one dissected with commercial thread and the other with Smartwire-01. The technique was standardized by keeping the entry point at the middle of the palm and the exit point at just medial to the palmaris longus tendon. The Numeric Rating Scale and Boston Carpal Tunnel Syndrome Questionnaire (BCTQ) were used to assess monthly outcomes for 6 months following the procedure. The Wilcoxon signed rank test and the Mann-Whitney-U test were performed to analyze the above variables in the two groups.

**Results:**

There was no definite evidence that the two groups have significant differences for any of the surveyed variables. The TCTR procedure with our newly developed thread also had significant improvements for all variables, showing its effectiveness in both pain and functional ability. The NRS and BCTQ severity and functional scales showed significant decreases just after the dissection and progressive improvement during each monthly follow-up of our study until the last assessment at 6 months.

**Conclusion:**

The study suggests that, our newly developed thread is as safe and effective as the commercial thread in TCTR, we therefore recommend a randomize controlled trial with above methodology.

## Introduction

Median nerve entrapment is the most common compressive peripheral neuropathy in the upper extremity, which is also well known as carpal tunnel syndrome [1]. The carpal tunnel has a transverse carpal ligament (TCL) as a roof and contains digital flexor tendons and median nerve. This space-limited osteo fibrous canal can lead to compression of the median nerve with idiopathic origin [2]. For diagnosis, clinical assessment is still considered to be the gold standard, while electrophysiological assessment is also very sensitive [3]. The Boston Carpal Tunnel Syndrome Questionnaire (BCTQ) is a validated patient-centered measure that quantifies symptoms and disability [4].

Various non-surgical treatments went through randomized controlled trials (RCTs). Local steroid injection is the most common treatment, but evidence for its effectiveness in halting disease progression is limited [5]. Three-quarters of patients with injections had disease progression, leading to surgical treatment. Surgical treatment, which releases the transverse carpal ligament, is considered the most effective treatment to remedy the symptoms [2]. To shorten the postoperative recovery period and reduce scars, the endoscopic surgical technique was introduced, but the cost and higher rate of nerve damage were the drawbacks [6]. The literature shows that the long-term effectiveness and recovery of nerve conduction were more effective with surgical treatment compared to various non-surgical treatments [7, 8].

The ultrasonography-guided thread carpal tunnel release (TCTR) technique was first proposed by Danqing Guo and his colleagues [9]. TCTR is a technique with the advantages of both surgical and non-surgical treatments, as transection of the transverse carpal ligaments is performed with minimal invasion to the patient, requiring only two percutaneous needle punctures. Real-time, high-resolution images of the anatomical structures around the carpal tunnel during the procedure could be gathered through ultrasonography imaging. TCTR’s effectiveness and safety were also confirmed by another group [10]. One study showed its superiority in a shorter return to work compared to open carpal tunnel release, although both were effective [11]. TCTR studies conducted by Danqing Guo et al. used a commercial thread (Loop & Shear, 0.23mm in diameter; Ridge & Crest Company, Monterey Park, California) [9, 12, 13]. Domestic dissecting threads (Smartwire-01, 0.27mm, Seoul, Korea) were developed for the percutaneous dissecting thread technique for better visibility under ultrasound and a higher cutting force within this technique.

This clinical study was conducted to propose the effectiveness of the newly developed thread by comparing it with the existing commercial thread.

## Materials and methods

This study was approved by the Institutional Review Board of Incheon St. Mary’s Hospital (OC21RADI0099). The need for consent was waived by the ethics committee.

### Participants

**Fig 1:**
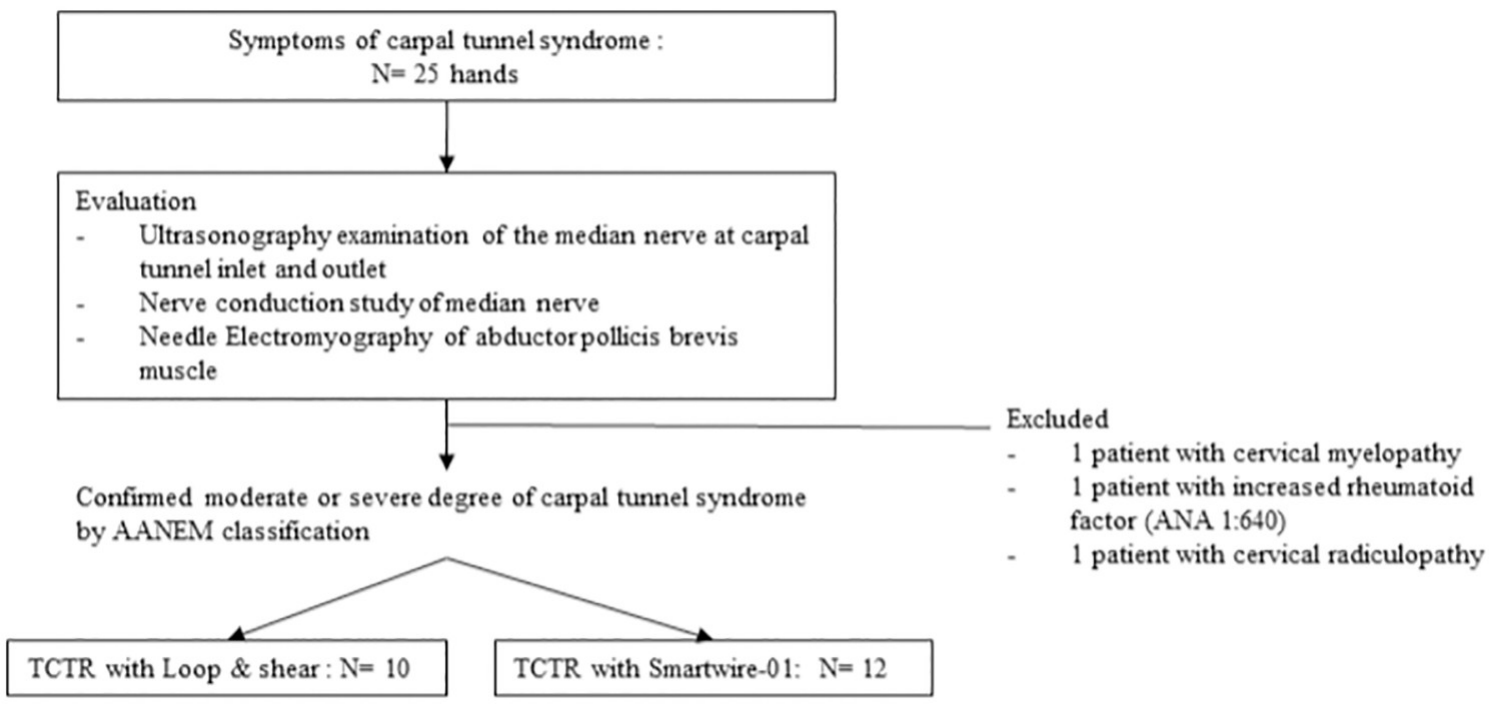
Flowchart detailing patient enrollment. Abbreviations: TCTR, thread carpal tunnel release.

We collected medical record of a total of 22 TCTR procedures which have been performed on 19 patients by one physiatrist from 2018.03.01 to 2021.06.30 (mean age = 53.8 years, standard deviation [SD] ± 12.8 years). Selection criteria included age >30 years to increase the unity of disease etiology into repetitive maneuvers using wrist. Symptoms that could clinically indicate carpal tunnel syndrome including hand numbness, tingling sensation, pain or weakness within median nerve-innervated dermatomes and myotomes, and positive signs with provocation tests, such as the Tinel sign at the wrist and Phalen’s test. Patient evaluation was conducted with both an ultrasonography and electrophysiological assessment. We excluded patients with comorbidities that could affect the result, such as cervical myelopathy, cervical radiculopathy, and increased rheumatoid factors. The two populations had similar characteristics, although more patients with a lower severity by the nerve conduction study were assigned to the domestic thread group. Ten patients were transected with the commercial thread (mean age = 54 years, moderate/severe 1/9, male/female 2/8, right/ left 5/5), while 12 patients were transected with our newly developed thread (mean age = 53 years, moderate/severe 6/6, male/ female 2/10, right/ left 6/6) (see Fig 1 and Table 1).

**Table 1:** General characteristics of hands.

Characteristics	Dissected with Loop & Shear^™^ (N = 10)	Dissected with Smartwire-01(N = 12)	P*
Age at patients	34 years old- 69 years old	31 years old– 73 years old	>.05
	(Mean = 54, SD = 10.97)	(Mean = 53, SD = 15.28)	
Degree (severe, %)	9 (90%)	6 (50%)	>.05
Sex (female, %)	8 (80%)	10 (80%)	>.05
Hand, right/left	5/5	6/6	>.05

Values are presented as number or percentage.

* Mann-Whitney U test was performed to compare characteristics of two groups.

### Procedure

**Fig 2:**
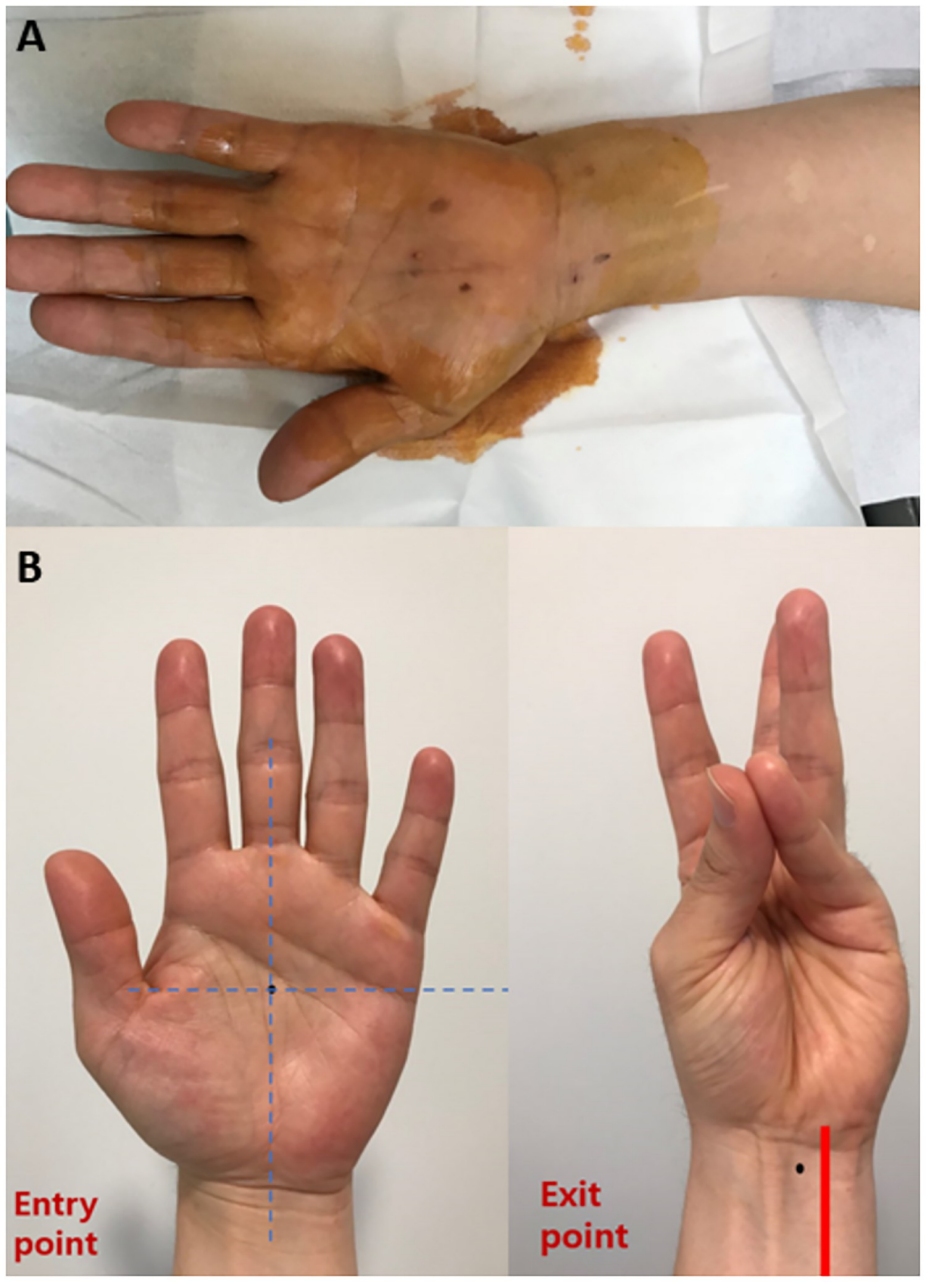
Standardized technique to determine entry and exit points. A: Patients’ upper extremity positioned on an arm board. B: Standardizing methods when marking entry and exit points.

**Fig 3:**
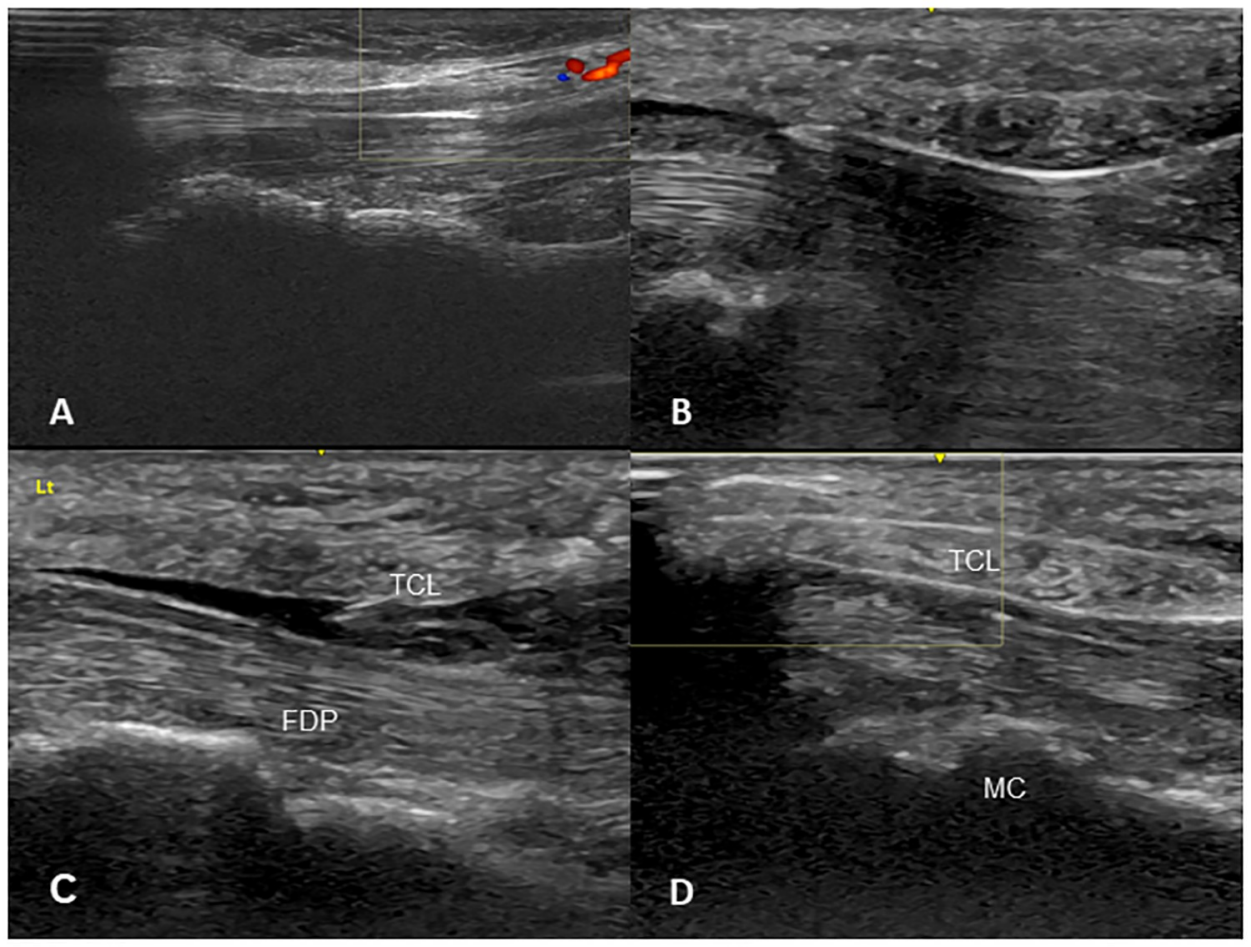
Ultrasound images of the carpal tunnel visualized during TCTR procedure. A: Anatomical landmark “duck’s beak”. B: Entry of 18G Tuohy epidural needle. C: Hydrodissection of carpal tunnel. D: Smartwire-01 looped around TCL. Abbreviations: TCL, transverse carpal ligament; MC, metacarpal bone; FDP, flexor digitorum profundus muscle.

TCTR was performed at ultrasound suite of our hospital. The patient’s upper extremity was positioned onto an arm board in a supine position with wrist slightly extended. The entry point was placed at the middle of the palm, just proximal to the superficial palmar arch (SupPA), where the central line of the 3^rd^ digit and a horizontal line from the apex of the interdigital fold between the thumb and index finger crosses perpendicularly. The exit point was placed just medial to the palmaris longus tendon, 1-2cm proximal to the distal wrist crease, which could be visualized by hand opposition of the thumb and 5^th^ digit with slight wrist flexion, in order not to damage the ulnar artery (Fig 2). All procedures were performed using an LOGIQ S7 ultrasound machine (GE Healthcare, Seoul, Korea) fitted with a ML6-15 (8 MHz) linear array transducer. With the ultrasound machine fitted, the patient’s structures were examined, and marked entry and exit points were confirmed. The “duck’s beak” described in previous studies, which is an anatomical landmark about 2mm in size used to locate the distal edge of the TCL and its relation with the palmar fat pad, was then identified. (Fig 3A) [14]. The modified TCTR technique suggested by Danquing Guo and colleagues was applied to each patient [9, 12, 13]. After local anesthesia with lidocaine, an 18G Tuohy epidural needle connected with 5ml water-filled syringe was used to guide the thread pathway. First, the needle, which was bent at a 30-degree angle 1cm distal to the tip and a 20-degree angle 4cm proximal to the tip, was inserted through the entry point at the palm, passing through the dorsal side of the TCL under the “duck’s beak” with ultrasound (Fig 3B). During needle insertion, hydrodissection was done to create an approximately 0.5cm diameter of safe space around the nerve by splitting connective tissue between the TCL and the median nerve (Fig 3C). After the needle tip exited at the determined exit point proximal to the TCL at the wrist, a thread was put through the needle. Leaving the thread at the dorsal side of the TCL, the needle was removed. Then, the second entry of the same Tuohy needle, unbent, was performed, passing through the palmar side of the TCL with ultrasound. Now the needle tip was directed above the “duck’s beak” between the palmar aponeurosis and the TCL. The second inserted needle was placed at the same longitudinal plane as the first insertion. Also, the exit point was at the same puncture hole to maintain the minimal invasive aspect of the technique. The thread left at the dorsal side of the TCL was then looped around the TCL through the needle (Fig 3D). Finally, the dissection of the TCL was performed by moving the thread reciprocally. After the removal of the thread, the patient’s dissected TCL was again visualized through ultrasonography.

### Materials

As carpal tunnel syndrome is a disease with a clinical diagnosis, we decided to measure subjective pain relief and functional improvement after the procedure. To standardize the effectiveness of TCTR in the two groups, we used the Numeric Rating Scale (NRS) and Boston Carpal Tunnel Syndrome Questionnaire (BCTQ), as they are both validated patient-centered measures for carpal tunnel syndrome patients in the literature. The NRS quantifies pain intensity using a 0–10 numeric rating scale. BCTQ scores range between 1 and 5, with 5 representing the most severe symptoms or functional limitation [15]. The outcomes were measured via phone interviews administered to patients pre-TCTR and at 1 day, 1 week, 2 weeks, 4 weeks, 2 months, 3 months, and 6 months after TCTR.

The commercial thread and the newly developed thread, Smartwire-01, consist of a similar chemical composition and wire structure. However, the maximal loading of the newly developed thread was 42.4N, which is greater than that of the commercial thread, 31.5N, and provides higher cutting force during the procedure (Table 2). Also, the Smartwire-01 has titanium nitride coating to improve sonographic visibility. Further assessment regarding these newly developed thread’s features would be done after this pilot study.

**Table 2:** General characteristics of threads.

Thread	Loop & Shear^™^	Smartwire-01
Chemical composition	Fe, Ni, Cr, Mo, Si, etc	Fe, Ni, Cr, Mo, Si, etc.
Material standard	STS316L	STS316L
Wire diameter	0.23 mm	0.27 mm
Maximum load	31.5 N	42.4 N

Abbreviation: Fe, Iron; Ni, Nickel; Cr, Chromium; Mo, Molybdenum; Si, Silicon; STS316L, Stainless steel 316 and L denotes the low content of carbon

### Statistical analysis

Medical record collected by a physiatrist was anonymized and then send to the data analyzer, unable to identify individual participants during or after data collection. The Wilcoxon signed rank test was performed to analyze pain reduction and functional gain over time in each group. (P<0.05) The Mann-Whitney U test was performed to compare the two groups at each outcome-measured period. (P>0.5).

## Results

**Fig 4:**
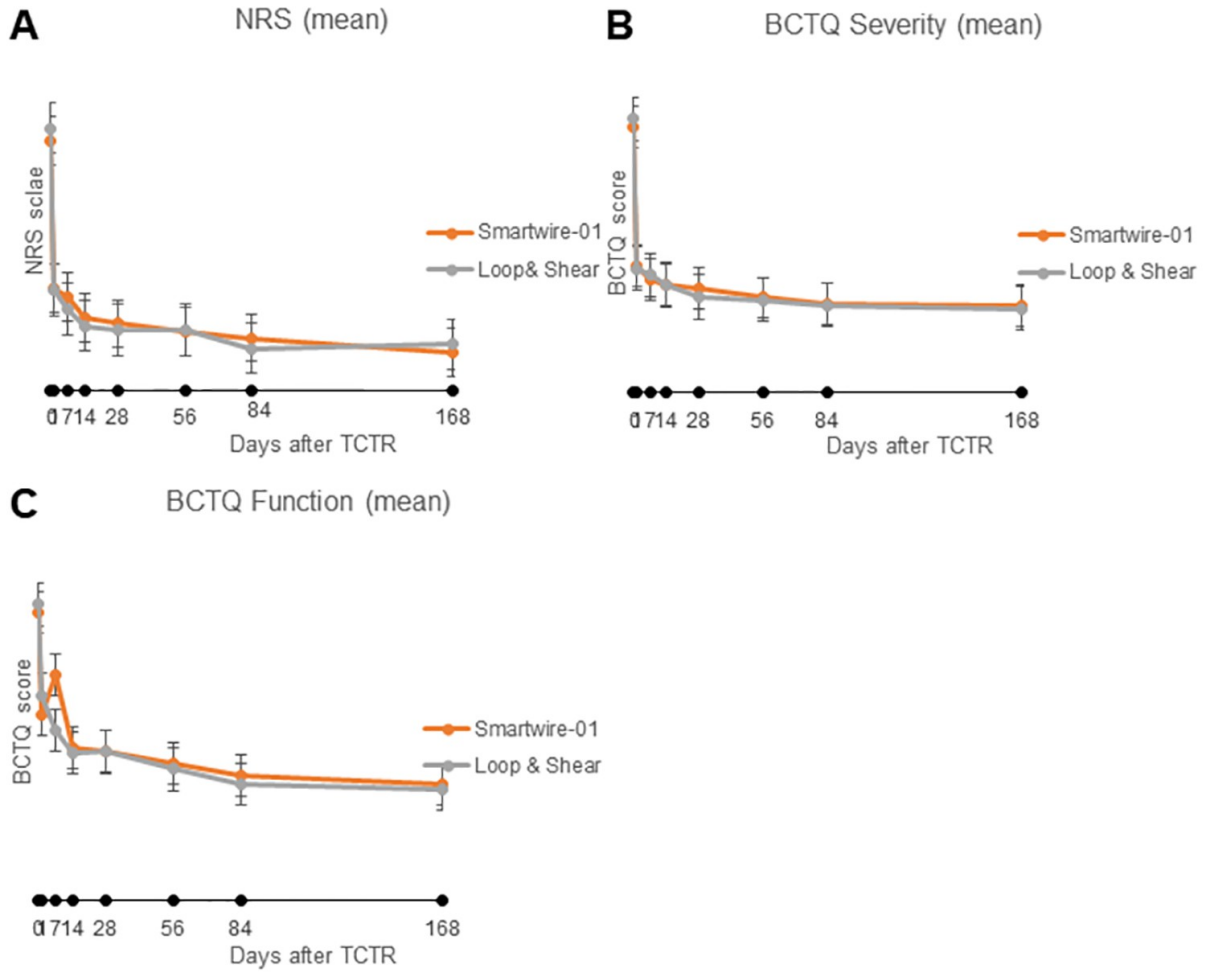
Post-TCTR time-dependent changes of pain and function of patients in the two threads (primary outcomes). A: NRS scale. B: BCTQ functional scale. C: BCTQ severity scale.

Ten hands using the Loop & Shear^™^ and 12 hands using the Smartwire-01 were included in the data analysis. The two groups showed similar tendencies in overall assessment. The two groups had no significant differences at pre-TCTR state. Before the procedure, patients in the Smartwire-01 group showed NRS score = 6.61 (SD ± 2.81), BCTQ function score = 3.23 (SD ± 0.92), and BCTQ severity score = 3.71 (SD ± 0.93), while patients in the Loop & Shear^™^ group showed NRS score = 6.95 (SD ± 1.98), BCTQ function score = 3.32 (SD ± 0.80), and BCTQ severity score = 3.83 (SD ± 0.81) (P>0.5). Both groups showed significant improvements in the primary outcome, pain, and severity just one day after the TCTR (P<0.05). Patients in the Smartwire-01 group showed NRS score = 2.69 (SD ± 1.44) and BCTQ severity score = 1.78 (SD ± 0.64), while those in the Loop & Shear^™^ group showed NRS score = 2.65 (SD ± 1.56) and BCTQ severity score = 1.74 (SD ± 0.46) (P>0.5). Following the initial effect, progressive improvement was shown until 2 weeks (P<0.05). At 2 weeks after the TCTR, patients in the Smartwire-01 group showed NRS score = 1.92 (SD ± 0.86) and BCTQ severity score = 1.52 (SD ± 0.43), while patients in the Loop & Shear^™^ group showed NRS score = 1.70 (SD ± 0.41) and BCTQ severity score = 1.51 (SD ±0.27) (P>0.5). Scores plateaued after 2–3 weeks, with continuous slight improvements for 6 months in both groups. At 6 months post-TCTR, the Smartwire-01 group showed NRS score = 1.00 (SD ± 0.82) and BCTQ severity score = 1.21 (SD ± 0.27), while the Loop & Shear^™^ group showed NRS score = 1.20 (SD ± 0.79) and BCTQ severity score = 1.18 (SD ± 0.11) (Fig 4A and 4B). Regarding hand function, patients’ functional improvement one day after the TCTR was not as clear as that of pain and severity but was present in both groups (P<0.05). At 2 weeks after the TCTR, the Smartwire-01 group showed BCTQ function score = 2.08 (SD ± 0.58), while the Loop & Shear^™^ group showed BCTQ function score = 2.30 (SD ±0.51) (P>0.5). Continuous improvement in patients’ hand function was seen in both groups. At 6 months post-TCTR, the Smartwire-01 group showed BCTQ function score = 1.30 (SD ± 0.33), while the Loop & Shear^™^ group showed BCTQ function score = 1.25 (SD ± 0.18) (Fig 4C). In the Smartwire-01 group, functional decline was observed at the 1^st^ week, scoring 2.08 (SD ± 1.56), but spontaneously recovered at the 2-week follow-up. Table 3 summarizes the course of pain reduction and functional recovery for 1 month post-TCTR. Table 4 summarizes monthly outcomes, assessing long-term effects of the TCTR on patients’ quality of living. A less dramatic but slowly continued improvement was shown from one month post-TCTR until the 6-month analysis in both groups (P<0.05). There was no definite evidence that newly developed thread was inferior to the commercial thread in long-term effectiveness between the two groups (P>0.5).

**Table 3:** Early recovery following TCTR: Mean (SD).

Outcome	Hand	Pre-TCTR	1day post	1week post	2 weeks post	4 weeks post	P*	P^¥^
**Pain** ^a^	New thread	6.61 (2.81)	2.69 (1.44)	2.46 (1.13)	1.92 (0.86)	1.76 (0.93)	< .05	>.05
	Control	6.95 (1.98)	2.65 (1.56)	2.15 (1.11)	1.70 (0.95)	1.60 (0.97)	< .05	
**Function** ^b^	New thread	3.23 (0.92)	2.08 (0.58)	2.53 (2.12)	1.70 (0.41)	1.66 (0.45)	< .05	>.05
	Control	3.32 (0.80)	2.30 (0.51)	1.90 (0.46)	1.65 (0.36)	1.66 (0.53)	< .05	
**Severity** ^c^	New thread	3.71 (0.93)	1.78 (0.64)	1.59 (0.35)	1.52 (0.43)	1.47 (0.37)	< .05	>.05
	Control	3.83 (0.81)	1.74 (0.46)	1.65 (0.31)	1.51 (0.27)	1.34 (0.19)	< .05	

^a^; Pain Numerical Rating Scale (0–10): 0 = no pain, 10 = worst pain,

^b^; Function by BCTQ (1–5): 1 = normal, 5 = abnormal,

^c^; Severity by BCTQ (1–5): 1 = no symptom, 5 = maximum symptoms.

* Wilcoxon signed rank test results

^¥^ Mann-Whitney U test results

**Table 4:** Long term recovery following TCTR: Mean (SD).

Outcome	Hand	Pre-TCTR	1 month post	2 months post	3 months post	6 months post	P*	P^¥^
**Pain** ^a^	New thread	6.61 (2.81)	1.76 (0.93)	1.54 (0.88)	1.38 (0.96)	1.00 (0.82)	< .05	>.05
	Control	6.95 (1.98)	1.60 (0.97)	1.60 (0.70)	1.10 (0.74)	1.20 (0.79)	< .05	
**Function** ^b^	New thread	3.23 (0.92)	1.66 (0.45)	1.53 (0.40)	1.40 (0.37)	1.30 (0.33)	< .05	>.05
	Control	3.32 (0.80)	1.66 (0.53)	1.47 (0.25)	1.30 (0.21)	1.25 (0.18)	< .05	
**Severity** ^c^	New thread	3.71 (0.93)	1.47 (0.37)	1.33 (0.33)	1.25 (0.30)	1.21 (0.27)	< .05	>.05
	Control	3.83 (0.81)	1.34 (0.19)	1.30 (0.19)	1.23 (0.15)	1.18 (0.11)	< .05	

^a^; Pain Numerical Rating Scale (0–10): 0 = no pain, 10 = worst pain,

^b^; Function by BCTQ (1–5): 1 = normal, 5 = abnormal,

^c^; Severity by BCTQ (1–5): 1 = no symptom, 5 = maximum symptoms.

* Wilcoxon signed rank test results

^¥^Mann-Whitney U test results

No adverse events were reported during the study. One patient with the Loop & Shear^™^ went through the TCTR again after 3 months due to a relapse of symptoms. By revisional TCTR, the patient had symptom relief for 6 months. No patients showed relapse in the Smartwire-01 group during the study. During the procedure, none of the Smartwire-01 threads was broken.

## Discussion

The objective of this study was to evaluate the effectiveness of the TCTR using the newly developed thread, Smartwire-01, compared to the commercial thread, Loop & Shear^™^. Just like previous studies in multiple groups, we also found that the TCTR procedure is effective and easily reproducible and can be further applied to South Korean patients. Our study suggests that newly developed thread is also effective in TCTR as in the commercial thread.

*Guo et al*. conducted the first clinical study introducing the modified TCTR in clinical settings, showing statistical significance of the TCTR’s efficacy regarding severity and function as described by the BCTQ, compared to previous studies conducted on open and endoscopic surgery [12]. The pain improvement course after TCTR suggested by Guo group was similar to our study, showing symptom and functional improvements in carpal tunnel syndrome patients occurred in the early phase after the procedure and showed slow improvement over the course of 12 months. The earlier improvement observed in pain and function was a significant advantage of this technique.

*Rojo-Manaute et al*. went through a randomized control study, showing that TCTR could lead to a faster return to daily living and work [14]. A similar tendency was shown in other groups, who independently went through the TCTR procedure [10]. These similar findings across several groups indicate that the modified TCTR is now a standardized technique which is reproducible by many trained physiatrists.

We applied standardized entry and exit points to all patients to offer strategies to ensure complete division of the transverse carpal ligament without damaging adjacent anatomical structures. The most common nearby structures that could be damaged during the CTR are the ulnar artery, which is often found radial to the hamate, and the superficial palmar arch of the transverse anastomosis between the ulnar and superficial radial arteries, that lies in a fat pad 5mm distal to the edge of the transverse carpal ligament [16]. Although complications including injury to other nerves in carpal tunnel release are quite rare [17], other surgical techniques besides the TCTR need superficial skin dissection, which could lead to damage of the palmar cutaneous branch of the median nerve. Sometimes, if the incision is taken too far distally, it could lead to common digital nerve damage [18]. Incisions induced painful scars that may also present after surgical release [19]. For surgical incision making, one group reported an anatomical safe space to avoid iatrogenic injury by palpating and measuring the distance between the flexor carpi radialis and flexor carpi ulnaris tendon [20]. Our entry and exit points described before are all located in this safe area.

In practice, as ultrasound identification of the locations of adjacent neurovascular structures was previously done before the TCTR, there were no cases with complications, although anatomical variations were present among patients. However, one study showed that even with trained experts, accuracy of blinded needle carpal tunnel injection may be less than previously believed [21]. As many previous cadaveric studies suggest [22], real-time ultrasound monitoring of the locations is necessary for reducing intraoperative and postoperative complications.

As seen in our study, the effectiveness and pattern of improvement in a patient’s subjective pain and function showed no statistically significant difference between two threads. Smartwire-01 with additional titanium coating is not only as effective but also has extra advantages which should be further studied compared to the commercial thread.

First, the thread could endure a higher maximum load due to reinforced loop tensile strength during TCTR. This makes Smartwire-01 to be stable during the technique. None of the Smartwire-01 threads broke during the procedure in this pilot study.

**Fig 5:**
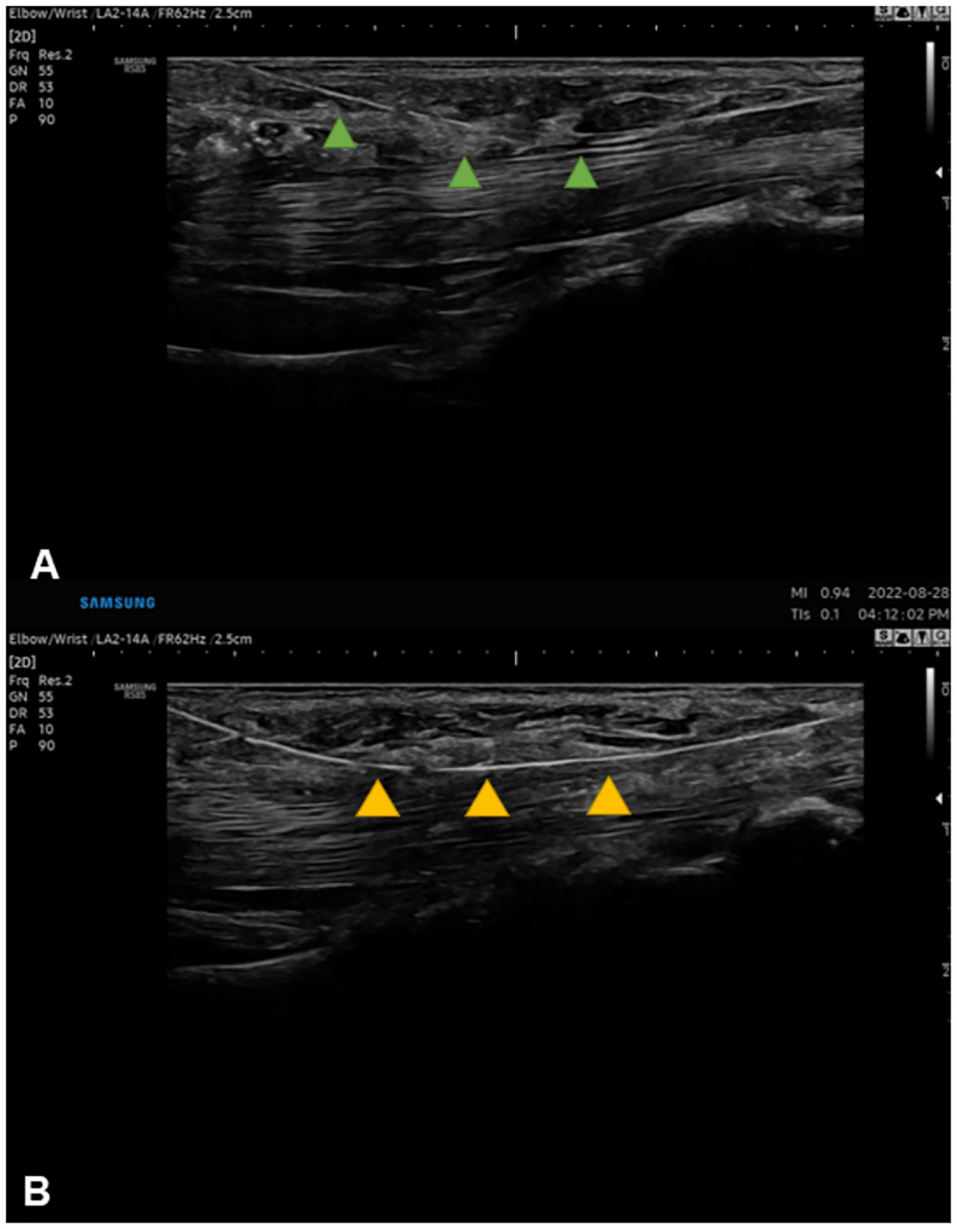
Ultrasound images of both threads in same settings. A, green arrows: Loop & Shear. B, yellow arrows: Smartwire-01.

**Fig 6:**
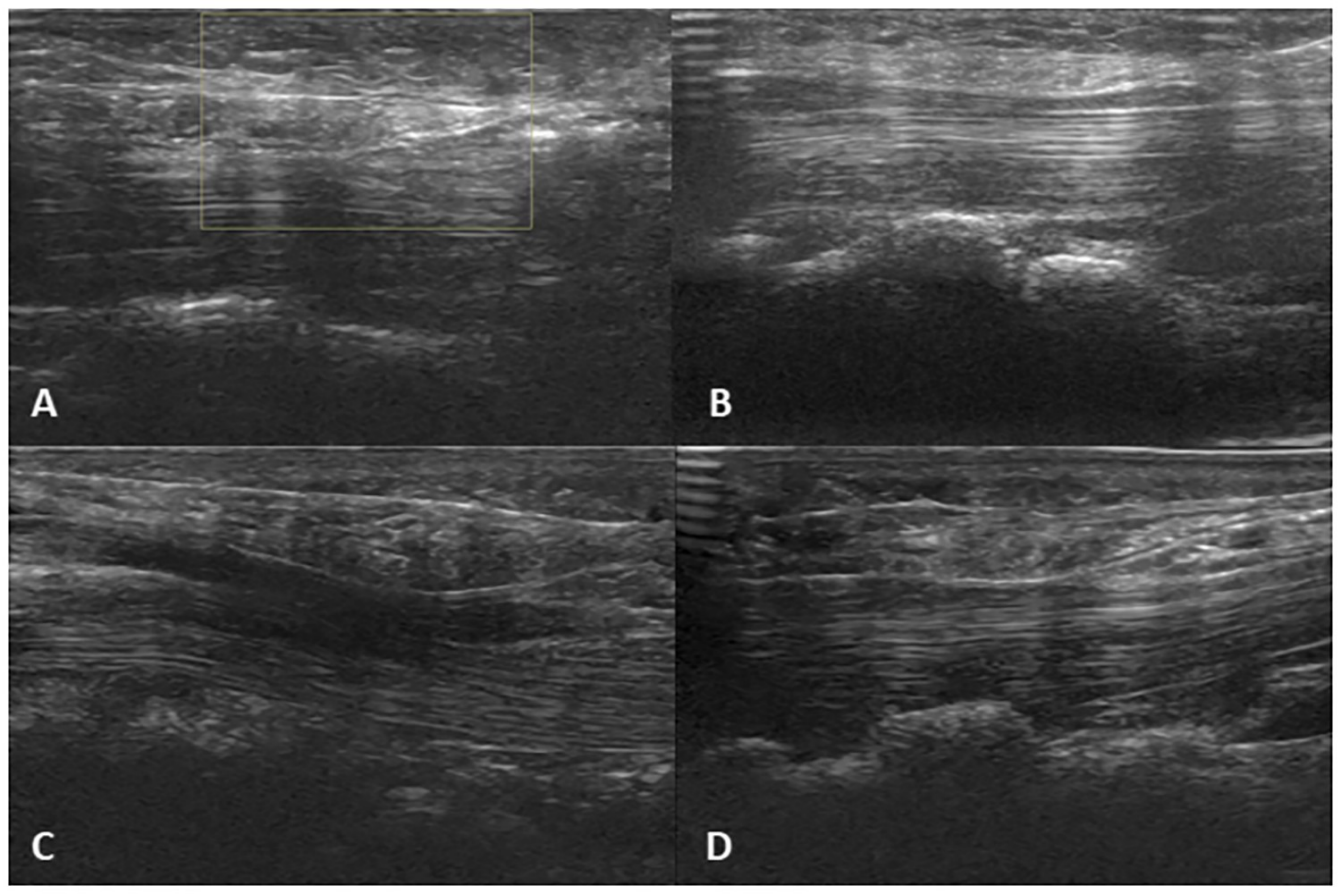
Realtime ultrasound images of both threads visualized during TCTR procedure. A, B: Loop & Shear thread. C, D: Smartwire-01. We could visualize the newly developed thread with higher acuity in ultrasonographic imaging compared to the commercial thread.

Second, this thin titanium nitride coating layer allows high visibility in ultrasound view. As identifying anatomical structures in the loop is crucial to avoid adverse events, clarity of the thread in ultrasound is important for standardizing TCTR. Smartwire-01 was visualized more clearly compared to the commercial thread in same ultrasonography settings. Fig 5 shows that Smartwire-01 has more hyperechogenic features overall and better clarity of margin compared to commercial one. This was also demonstrated in real-time ultrasonography imaging during TCTR technique (Fig 6).

Third, sufficient and adequate elasticity was provided, which makes the performer to handle the thread more easily. Adequate elasticity makes insertion of thread inside the Tuohy needle much easier, which is difficult part of the technique that requires training for physiatrists. The newly developed thread gives these additional benefits to the technique, but as which should be further assessed under experimental settings.

Failure of primary CTR is reported in 7%-25% of patients in previous case series, with approximately 5% to 12% requiring secondary surgery [19, 23]. Causes of treatment failure are categorized into three groups: incomplete release, recurrent compression, and incorrect diagnosis [18]. In this pilot study, one patient relapsed after TCTR. At initial procedure, this patient’s palmar aponeurosis was preserved, as modified Guo technique suggests [12]. However, as patient’s symptom recurred, revisional TCTR was done with additional palmar aponeurosis release and patient’s symptom was improved, lasting at least for 6 weeks. As we did not go through thorough study controlling different conditions regarding palmar aponeurosis release, it requires further studies to see whether palmar aponeurosis release affects improvement after TCTR.

The study was conducted by one physiatrist, and the standardized TCTR technique described above was applied to each patient, reducing other variables during the procedure. Assessment was performed by a blinded assessor after the patient’s information was deleted to provide credibility to the result.

The limitation of this study is the small number of patients enrolled in each group. Fifty-three for each group was calculated as adequate number to have high statistical meanings, but it was yet fulfilled in this pilot study [24]. Further studies with more patients are required in order to statistically assess low-incidence complications, as carpal tunnel release is known to cause median nerve injury in 0.06% and ulnar nerve injury in 0.03% of cases [17]. As more patients with a moderate degree of carpal tunnel syndrome were enrolled in the Smartwire-01 group, although there was no evidence for difference between the two groups when outcome measures were analyzed prior to TCTR, it might have led to better results in the Smartwire-01 group. Therefore, we need to further apply the TCTR to more patients and follow up for a longer period to better qualify the data. As the total number of enrolled patients was small, one physiatrist fully participated in all procedures to reduce other bias. In further studies, more physiatrists should enroll in the TCTR procedures using the Smartwire-01 to statistically analyze whether the stability of the thread has superiority over the commercial one. Besides the subjective patient measurements, studies including objective measurements such as grip strength and electromyography assessment could support the use of our newly developed thread. *Burnham et al*. showed objective improvement through TCTR through both electrodiagnostic study and cross-sectional area assessed by ultrasonography [10]. Further studies are required in the Smartwire-01 group.

Although the technique has advantages of both surgical and non-surgical treatment, surgery is still more preferred in the local clinic. To universalize the technique, development of this new thread with improved visual acuity in ultrasonography view, clear margins, and stability could lead many physiatrists to more easily access the TCTR.

## Conclusion

This pilot study suggests that our newly developed thread, Smartwire-01, is also effective in TCTR as the commercial thread, Loop & Shear, for pain relief and increasing functional activity. Therefore, additional studies with more samples should be performed.

## Supporting information

S1 File (PDF)
