# Supplementary material for: A clinical study of thread carpal tunnel release with a newly developed thread: A retrospective pilot study
Source: PLoS One. 2022 Oct 21;17(10):e0276630. doi: 10.1371/journal.pone.0276630 (PMC9586357; doi:10.1371/journal.pone.0276630)
Supplement: S1 File — (PDF) [file pone.0276630.s001.pdf]

No.	Gender	Age	Hand	Period	NRS	severity	function
**1**	**M**	**69**	**Rt**	0	7	4.32	4.00
				1day	4	2.25	3.00
				1wk	3	1.95	2.65
				2wk	2	1.34	2.12
				4wk	2	1.20	2.00
				8wk	2	1.20	1.58
				12wk	1	1.15	1.47
				26wk	2	1.20	1.35
**2**	**F**	**69**	**Lt**	0	6	4.55	4.00
				1day	2.5	2.27	3.00
				1wk	2.5	2.09	1.25
				2wk	1	1.91	1.25
				4wk	1	1.27	1.25
				8wk	2	1.25	1.50
				12wk	1	1.36	1.35
				26wk	1	1.25	1.35
**3**	**F**	**51**	**Rt**	0	5.5	4.09	3.38
				1day	2	2.04	2.80
				1wk	2	1.59	1.80
				2wk	1	1.35	1.30
				4wk	2	1.24	1.30
				8wk	1	1.35	1.38
				12wk	1	1.20	1.15
				26wk	1	1.25	1.20
**4**	**F**	**57**	**Lt**	0	8	3.09	3.75
				1day	4	1.91	2.65
				1wk	4	1.85	2.65
				2wk	3	1.78	2.13
				4wk	3	1.56	1.88
				8wk	2	1.56	1.88
				12wk	2	1.45	1.56
				26wk	2	1.36	1.48
**5**	**F**	**54**	**Rt**	0	5	2.91	2.52
				1day	4	1.58	2.05
				1wk	2	1.45	1.45
				2wk	2	1.36	1.37
				4wk	2	1.25	1.30
				8wk	2	1.20	1.30
				12wk	1	1.25	1.37
				26wk	2	1.25	1.20
**6**	**F**	**63**	**Rt**	0	4	2.36	2.13
				1day	4	2.00	2.10

				1wk	3	1.82	2.05
				2wk	3	1.64	1.98
				4wk	2	1.50	1.62
				8wk	2	1.50	1.58
				12wk	2	1.45	1.60
				26wk	2	1.20	1.55
**7**	**F**	**52**	**Lt**	0	10	4.82	4.50
				1day	3	1.81	2.00
				1wk	2	1.72	1.88
				2wks	1	1.65	1.75
				4wks	0	1.63	2.88
				8wks	2	1.55	1.25
				12wk	2	1.23	1.25
				26wk	1	1.15	1.20
**8**	**F**	**52**	**Rt**	0	7	3.55	3.25
				1day	3	1.54	1.97
				1wk	2	1.45	1.85
				2wks	2	1.40	1.55
				4wks	2	1.35	1.45
				8wks	2	1.25	1.64
				12wk	1	1.25	1.30
				26wk	1	1.20	1.15
**9**	**M**	**34**	**Lt**	0	7	4.27	2.25
				1day	0	1.00	1.50
				1wk	0	1.00	1.50
				2wks	0	1.00	1.22
				4wks	0	1.00	1.05
				8wks	0	1.00	1.00
				12wk	0	1.00	1.00
				26wk	0	1.00	1.00
**10**	**F**	**49**	**Lt**	0	10	4.36	3.50
				1day	0	1.00	2.02
				1wk	1	1.64	1.95
				2wks	2	1.64	1.88
				4wks	2	1.45	1.88
				8wks	1	1.09	1.63
				12wk	0	1.00	1.00
				26wk	0	1.00	1.00
**11**	**F**	**49**	**Rt**	0	10	3.64	3.25
				1day	2	1.27	1.55
				1wk	2	1.18	1.45
				2wks	1	1.18	1.34
				4wks	1	1.00	1.13

				8wks	1	1.00	1.12
				12wk	1	1.00	1.12
				26wk	1	1.00	1.12
**12**	**F**	**64**	**Lt**	0	2	1.91	1.00
				1day	2	1.00	1.00
				1wk	1	1.00	1.00
				2wks	1	1.00	1.00
				4wks	0	1.00	1.00
				8wks	0	1.00	1.00
				12wk	0	1.00	1.00
				26wk	0	1.00	1.00
**13**	**F**	**57**	**Lt**	0	7	4.63	3.13
				1day	3	2.18	2.00
				1wk	3	2.00	1.88
				2wks	2	2.00	1.76
				4wks	2	1.85	1.64
				8wks	2	1.76	1.58
				12wk	2	1.50	1.52
				26wk	1	1.24	1.22
**14**	**M**	**67**	**Rt**	0	6	3.36	2.25
				1day	3	1.36	1.65
				1wk	3	1.25	1.50
				2wks	2	1.00	1.50
				4wks	2	1.45	1.56
				8wks	2	1.25	1.45
				12wk	2	1.25	1.25
				26wk	2	1.20	1.00
**15**	**M**	**31**	**Rt**	0	4	3.55	3.00
				1day	3	3.27	3.00
				1wk	3	1.91	2.94
				2wks	3	2.27	2.60
				4wks	3	2.18	2.62
				8wks	3	2.05	2.43
				12wk	3	1.88	2.22
				26wk	3	1.80	2.15
**16**	**F**	**66**	**Rt**	0	8	4.45	4.75
				1day	4	2.05	2.46
				1wk	3	1.88	2.20
				2wks	2	1.64	1.64
				4wks	2	1.70	1.62
				8wks	2	1.52	1.58
				12wk	2	1.30	1.44
				26wk	1	1.25	1.46

**17**	**F**	**66**	**Lt**	0	8	4.02	3.88
				1day	5	2.25	1.87
				1wk	4	2.05	1.85
				2wks	3	1.86	1.66
				4wks	3	1.75	1.66
				8wks	2	1.42	1.45
				12wk	2	1.25	1.50
				26wk	1	1.25	1.45
**18**	**F**	**49**	**Rt**	0	10	4.32	3.65
				1day	3	1.56	1.87
				1wk	3	1.40	1.64
				2wks	2	1.20	1.50
				4wks	2	1.20	1.45
				8wks	1	1.15	1.23
				12wk	1	1.00	1.15
				26wk	1	1.00	1.15
**19**	**F**	**35**	**Lt**	0	8	4.45	3.50
				1day	3	2.05	2.12
				1wk	3	1.85	1.95
				2wks	2	1.95	1.77
				4wks	2	1.72	1.65
				8wks	2	1.65	1.62
				12wk	1	1.75	1.65
				26wk	1	1.72	1.52
**20**	**F**	**49**	**Lt**	0	7	3.91	3.63
				1day	4	1.91	2.52
				1wk	3	1.52	2.13
				2wks	3	1.24	1.56
				4wks	2	1.15	1.43
				8wks	2	1.04	1.20
				12wk	2	1.00	1.15
				26wk	1	1.00	1.20
**21**	**F**	**73**	**Rt**	0	3	1.91	3.13
				1day	3	1.54	3.00
				1wk	3	1.45	2.85
				2wks	2	1.30	2.24
				4wks	2	1.25	2.32
				8wks	2	1.25	2.04
				12wk	2	1.25	1.85
				26wk	1	1.20	1.52
**22**	**F**	**34**	**Lt**	0	3	3.45	1.63
				1day	0	1.09	1.42
				1wk	0	1.18	1.39

2wks	0	1.18	1.37
4wks	0	1.18	1.37
8wks	0	1.09	1.37
12wk	0	1.09	1.37
26wk	0	1.09	1.20
